# Editorial: Case reports in schizophrenia and psychotic disorders

**DOI:** 10.3389/fpsyt.2023.1282780

**Published:** 2023-09-13

**Authors:** Massimo Tusconi, Gabriele Nibbio, Rishab Gupta, Erika Carr

**Affiliations:** ^1^Section of Psychiatry, Department of Medical Sciences and Public Health, University of Cagliari, Cagliari, Italy; ^2^Department of Clinical and Experimental Sciences, University of Brescia, Brescia, Italy; ^3^Brigham and Women's Faulkner Hospital and Harvard Medical School, Boston, MA, United States; ^4^Department of Psychiatry, Yale University School of Medicine, New Haven, CT, United States

**Keywords:** schizophrenia, case reports, psychosis, catatonia, encephalitis, treatment resistant psychosis

The publication of case reports is essential within scientific literature in order to highlight data that provide essential information for disease management, to bring to attention atypical disease presentations, rare adverse effects of treatments, and to generate new hypotheses. Based on epistemological insights, it emerges that the idea that an observation can be reflected in a scientific discovery led to the issue of induction vs. deduction (1). Karl Popper, the master of deductive thinking, theorized a hypothetico-deductive scheme of scientific reasoning, according to which random observations assume relevance on the basis of their unexpected characteristics, posing as a refutation of our previous beliefs and paving the way for new conjectures and theories (2).

On these fundamentals, it follows that it is not always possible to identify all the salient aspects of diseases through systematic clinical studies. Sometimes, case reports can trigger research processes that delve into pathophysiologic mechanisms underlying various diseases (3). It has been said that “case reports and case series may be at the lowest or weakest level of clinical evidence, but they often remain the first line of evidence this is where it all begins (4).”

Schizophrenia is considered one of the most severe psychiatric disorders (5). It is often associated with significant neurocognitive and social cognition deficits (6–8), daily functional impairment for many, high levels of internalized stigma (9, 10), and poor real-world outcomes (11–13). In this context, case reports and case series of people living with schizophrenia and/or other psychotic disorders are of considerable interest in both research and clinical settings, and as a mechanism to build lives of meaning as people with the experiences define.

The goal of our Research Topic was to highlight rare and special characteristics of the psychosis spectrum by reinforcing emerging aspects and new therapeutic approaches with a hope to achieve an optimal outcome for patients with psychosis-spectrum disorders.

In a paper by Karpov et al. describing Neuroleptic Malignant Syndrome in an individual with Williams Syndrome (WS), the authors described the appearance of somatic delusions in a patient with severe anxiety and concerns about a headache. The authors hypothesized correlation between psychotic onset and severe anxiety.

In another case report, Pavăl et al. observed that despite using a combination of haloperidol and olanzapine, the patient with Anti-N-Methyl-D-Aspartate Receptor (anti-NMDAR) Encephalitis did not experience intolerance to antipsychotics, contrary to what is typically seen in cases of anti-NMDAR encephalitis. This was an unusual case in which psychiatric symptoms occurred at the onset of illness, seen in <1% of patients with anti-NMDAR encephalitis. The patient had negative MRI brain, EEG, and CSF studies.

Baek et al. delved into analysis of recovery from hallucinations and cognitive impairment with the use of donepezil in a patient with schizophrenia in the setting of carbon monoxide (CO) poisoning. The patient underwent hyperbaric oxygen therapy without improvement in delayed neurological sequelae (DNS) of CO poisoning. Subsequently, cognitive function dramatically improved with the Donepezil trial. In the case, the patient's persistent (despite adequate pharmacotherapy) severe auditory hallucinations, present before the suicide attempt involving CO poisoning, also improved remarkably after initiation of donepezil.

In another report, Miller et al. highlighted the connection between psychotic symptoms requiring hospitalization and Delta-8-THC (Tetrahydrocannabinol) use in three individuals. Of note, Delta-8-THC's psychiatric effects are not fully described in the literature. The authors suggest the need for more regulation of hemp derivatives, as well as confirmatory classification by governments through the appropriate agencies.

Jin et al. illustrated a fascinating case of schizophrenia comorbid with antiphospholipid syndrome (APS), beta-thalassemia, and monoclonal gammopathy of undetermined significance (MGUS). The patient was treated with chlorpromazine for schizophrenia and developed Lupus Anticoagulant antibody and APS. The serum protein electrophoresis found monoclonal IgA peak, leading to a diagnosis of MGUS. In addition, beta-thalassemia with CD41-42 genotype was also diagnosed. This condition is extremely rare, especially in patients with schizophrenia and APS.

Finally, Liebrand et al. characterized psychosis and catatonia in an adolescent with adipsic hypernatremia with autoantibodies against the subfornical organ. They key features of this condition which might guide clinicians are: childhood-onset complex and treatment-resistant psychosis and catatonia in combination with severe behavioral problems, fatigue, absent thirst, hypernatremia, and hyperprolactinemia.

In summary, this Research Topic has shown that careful and thorough use and evaluation of case reports can bring to light unexamined aspects of diseases not evaluated in systematic studies. This approach can pose as a fundamental point of research, especially in those areas where knowledge is not fully dissected due to the vastness and variety of phenomenology and psychiatric disorders. Delineating new facets through clinical cases could stimulate new clinical and laboratory studies to clarify patterns that are currently not fully described, eventually enabling better understanding and clinical management of diseases.

## Author contributions

MT: Writing—original draft, Writing—review and editing. GN: Writing—original draft, Writing—review and editing. RG: Writing—original draft, Writing—review and editing. EC: Writing—original draft, Writing—review and editing.
